# *Salmonella* Typhimurium gastroenteritis leading to chronic prosthetic vascular graft infection

**DOI:** 10.1099/jmmcr.0.005104

**Published:** 2017-08-08

**Authors:** Milo Cullinan, Michael Clarke, Tim Dallman, Steven Peart, Deborah Wilson, Daniel Weiand

**Affiliations:** ^1^​ Freeman Hospital, Freeman Road, Newcastle Upon Tyne NE7 7DN, UK; ^2^​ Public Health England, 61 Colindale Ave, London NW9 5EQ, UK; ^3^​ Public Health England, Citygate Gallowgate, Newcastle Upon Tyne NE1 4WH, UK

**Keywords:** Chronic prosthetic vascular graft infection (PVGI), *Salmonella* Thyphimurium infection, gastroenteritis, cutaneous abscess, removal of the prosthetic vascular graft, intravenous piperacillin-tazobactam

## Abstract

**Introduction.** It is estimated up to 6 % of prosthetic vascular grafts become infected. *Staphylococcus aureus* is predominant in early infection and coagulase-negative staphylococci are predominant in late infections. *Enterobacteriaceae* cause 14–40 % of prosthetic vascular graft infections. This is, to our knowledge the first reported case of *Salmonella* gastroenteritis causing chronic prosthetic vascular graft infection (PVGI).

**Case presentation.** A 57 years old lady presented with signs and symptoms of prosthetic vascular graft infection. Three years earlier, she had undergone a prosthetic axillo-femoral bypass graft for critical limb ischaemia. The infected prosthetic vascular graft was removed and *Salmonella* Typhimurium was isolated on culture. In the intervening period, *Salmonella* Typhimurium was isolated from a faecal specimen, collected during an episode of acute gastroenteritis. Whole-genome sequencing (WGS) showed that the respective *Salmonella* Typhimurium isolates differed by only a single nucleotide polymorphism (SNP). *Salmonella* Typhimurium was not isolated on culture of a faecal specimen collected five days following cessation of antimicrobial therapy. Six months after removal of the prosthetic graft, the patient remains under follow-up for her peripheral vascular disease, which currently requires no further surgical intervention.

**Conclusion.** This case has clear implications for the management of chronic PVGI. It is vital to collect high-quality surgical specimens for microbiological analysis and empirical choices of antibiotics are unlikely to cover all potential pathogens. It may also be prudent to enquire about a history of acute gastroenteritis when assessing patients presenting with chronic PVGI.

## Abbreviations

API 20E, Analytical Profile Index 20E; COPD, Chronic obstructive pulmonary disorder; CRP, C-reactive protein; CT, Computed tomography; GP, General practitioner; MALDI–TOF, Matrix assisted laser desorption/ionisation–time of flight; PVGI, Prosthetic vascular graft infection; SNP, Single nucleotide polymorphism; SRS, Salmonella reference service; WCC, White cell count; WGS, Whole-genome sequencing.

## Introduction

It is estimated that 0.5–6 % of prosthetic vascular grafts become infected [1]. *Staphylococcus aureus* is predominant in early infection and coagulase-negative staphylococci are predominant in late infections. *Enterobacteriaceae* cause 14–40 % of prosthetic vascular graft infections. To date, only seven cases of prosthetic vascular graft infection (PVGI) involving species of the genus *Salmonella* have been reported [2]. However, none of these cases presented with a history of gastroenteritis. This is, to our knowledge, the first reported case of *Salmonella* gastroenteritis causing chronic PVGI.

## Case report

In December 2012, a 54 years old female presented with critical limb ischaemia and imaging revealed extensive aorto-iliac occlusive disease. Limb salvage was achieved by means of an extra-anatomic axillo-bifemoral bypass, as the extent of the disease was considered unsuitable for endovascular intervention. A Gelsoft gelatin-impregnated knitted vascular prosthesis was used and she received clindamycin and gentamicin as prophylaxis for the procedure, as per local guidelines.

Underlying comorbidities included Type 2 diabetes (controlled with Metformin), and severe chronic obstructive pulmonary Disorder (COPD). In 2006, the patient suffered an out-of-hospital cardiac arrest due to an infective exacerbation of her COPD, and required a prolonged admission on intensive care. Medications prescribed to control her COPD included: Budesonide and Folmeterol combination inhaler; Tiotropium; Theophyline (Uniphyline Continus); and Montelukast. The patient’s history of severe COPD made her unsuitable for open aortic surgery.

In July 2014, the patient developed a diarrhoeal illness, which followed a week-long history of malaise, anorexia and feverish symptoms. She suffered watery diarrhoea four to five times a day, but with no associated abdominal pain, and no blood in her stools. She was admitted to her local hospital where she was treated with intravenous crystalloid until her diarrhoea resolved spontaneously. Culture of a faecal specimen led to the isolation of *Salmonella* Typhimurium. An environmental health assessment concluded that she had not visited any restaurants, saying that she always prepared her own food. She was unemployed, and had no pets. The patient also denied having ever travelled abroad, and had not recently left the North East of England, where she lives in a coastal town.

In February 2015, the patient presented to her general practitioner (GP) with a five-month history of painful lump in her left axilla. On examination, the lump was approximately 3 cm in diameter, erythematous, tender and fluctuant, and the GP diagnosed a simple abscess. The GP prescribed oral antibiotic therapy, but this had very limited clinical effect. Therefore, the decision was made to lance the presumed abscess at a local hospital. Only a minimal amount of serosanguinous fluid was evacuated, and no unequivocal pathogens were isolated from a wound swab sent for culture. Following this intervention, the presumed abscess was regularly dressed in the community by a team of district nurses. However, the patient reported a green discharge, sufficient to saturate the dressing on a daily basis. The lump became progressively more painful, despite opiate analgesia, and the patient was eventually unable to abduct her left arm.

In October 2015, the patient was referred to the vascular surgical outpatient clinic. On examination, there was a 0.5 cm ulcer over the lower margin of the left lateral thorax, although there was little in the way of surrounding inflammation. Computed tomography (CT) showed that the lesion on the chest wall clearly communicated with the underlying prosthetic axillo-bifemoral bypass graft. The graft remained fully patent, and the axillary and bilateral femoral anastomoses appeared intact. A diagnosis of low-grade PVGI infection was made. In view of the patient’s multiple comorbidities and limited options for further revascularisation, surgical intervention with prosthetic graft removal was considered high-risk, and a decision was taken to continue with conservative management of the PVGI. At this stage, *Staphylococcus aureus* and *Streptococcus dysgalactiae* were cultured from a superficial wound swab. Both organisms were susceptible *in vitro* to flucloxacillin. The patient was commenced on oral flucloxacillin and, by February 2016, the patient’s wound discharge was minimal, with full range of movement of her left arm.

**Fig. 1.**
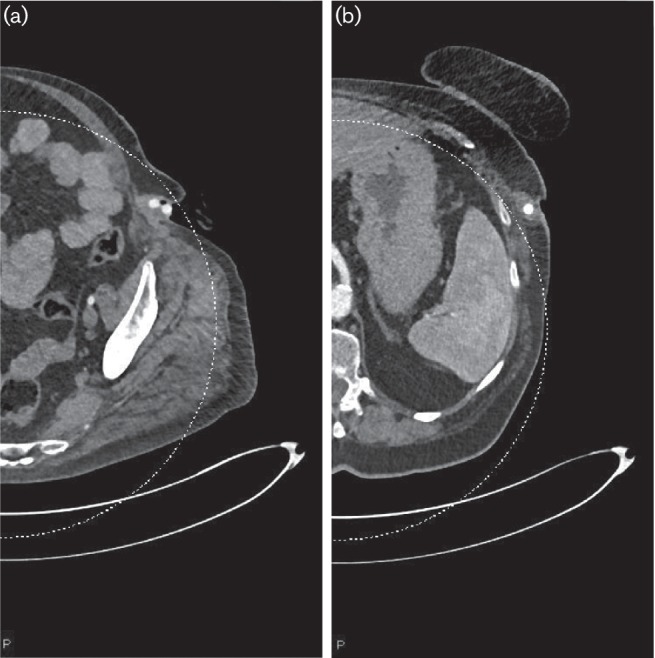
Axial images of a CT angiogram of the whole aorta and lower limbs showing (a) the graft exposed in the left groin and (b) an area of fluid or soft tissue attenuation surrounding the graft in the left axilla.

The patient’s clinical condition remained stable until September 2016 when she presented with acute-onset pain in the left groin. On examination, there was a large, discharging ulcer in her left groin, measuring approximately 4 cm by 2 cm with prosthetic graft clearly visible in its base. White cell count (WCC) was 15.49×10^9^ l^−1^ and her C-reactive protein (CRP) was 32 mg l^−1^. CT angiogram showed an area of fluid or soft tissue attenuation around the graft, in the left axilla, and confirmed that the graft was exposed in the left flank and groin (Fig. 1).

In September 2016, it was clear that there was no option other than to remove the graft due to the risk of life-threatening haemorrhage, but that consideration also needed to be given to maintaining lower limb perfusion. Via bilateral percutaneous femoral access, a 14 mm nitinol stent was placed in the aorta and 'kissing’ nitinol stents then placed down to the left common iliac artery and the right external iliac artery. The efficacy of the stents was then confirmed by temporarily tying off the exposed inguinal graft. This caused no symptoms suggestive of ischaemia in her legs. Subsequently, the infected prosthetic axilla-bifemoral graft was completely removed, using clindamycin and gentamicin as surgical prophylaxis.

## Investigations

Tissue and the explanted graft collected at the time of removal of the infected prosthetic axilla-bifemoral bypass graft were cultured on cysteine lactose deficient (CLED) agar for at least 18 h at 36 °C in ambient air. A non-fermenting coliform was identified as a species of Salmonella by matrix assisted laser desorption/ionisation–time of flight mass spectrometry (MALDI–TOF). This was confirmed with an Analytical Profile Index 20E (API20E) and serological agglutination testing identified it as *Salmonella enterica* serotype Typhimurium. This was confirmed by the Salmonella Reference Service, Colindale (SRS).

Antimicrobial susceptibility testing using the EUCAST disk diffusion method demonstrated resistance to amoxicillin and cefuroxime. Salmonella is intrinsically resistant to clindamycin and gentamicin is unlikely to be effective *in vivo*.

Whole-genome sequencing (WGS) showed that the *Salmonella* Typhimurium isolated from faeces, in 2014, and the infected prosthetic vascular graft, in 2016, differed by only one single nucleotide polymorphism (SNP). The SNP address profile has only been seen in these two isolates out of 5000 other genomes tested at the reference laboratory (Personal communication, T. Dallman, SRS, Colindale). The difference of one SNP between the isolates is well within what would be expected over this time frame. *Salmonella* Typhimurium mutation rates are estimated at 1–10 SNPs per year [3].

## Outcome and follow-up

The patient was prescribed a five-day course of intravenous piperacillin–tazobactam, to which the *Salmonella* Typhimurium tested susceptible. Two days post-operatively, the patient noticed improvement of her pain. WCC reduced to 9.39×10^9^ l^−1^ and CRP reduced to 5 mg l^−1^. Her opiate requirements fell from 40 mg daily, on admission, to 10 mg daily.

Seven days after removal of the infected prosthetic vascular graft, the patient was discharged home. She had completed five days of intravenous piperacillin–tazobactam, and a decision was taken to cease antimicrobial therapy. The surgical sites were satisfactory in appearance.

Three weeks post-operatively, the patient was seen in the vascular surgical outpatient clinic. She was able to walk into the clinic, and the surgical sites had healed completely. It was noted that *Salmonella* Typhimurium was not isolated on culture of a faecal specimen collected ten days post-operatively, following cessation of antimicrobial therapy.

Six months post-operatively, the patient remains under follow-up for her peripheral vascular disease, which currently requires no further surgical intervention.

## Discussion

This is, to our knowledge, the first reported case of *Salmonella* gastroenteritis causing chronic PVGI. The results of WGS support our theory that haematogenous spread of *Salmonella* Typhimurium either during the episode of acute gastroenteritis, or because of subsequent translocation from chronic carriage, led to bacterial ‘seeding’ of the prosthetic graft. Once infected, the resulting chronic inflammatory process led to the infected prosthetic axilla-bifemoral graft eroding through the skin, in both the axillary and inguinal regions.

On reviewing the wider literature, there have been seven reported cases of *Salmonella* species causing PVGI (See Table 1). In one case, the prosthetic graft was placed in a patient with an external iliac aneurysm that was later felt to have been caused by *Salmonella* arteritis [4]. In another case, the graft had eroded into the bowel providing a route of direct spread [5]. In all other cases, the primary source of infection remained unclear [2, 6–9].

**Table 1.** Summary of published cases of *Salmonella* PVGI

Source	Graft	Presumed Source	Sub-speciation
[8]	Aorto-femoral graft	None identified	*S*. Cholerasuis
[5]	Aortic graft	Enteric erosion of prosthetic vascular graft	Not reported
[4]	Ilio-femoral graft	Pre-existing *Salmonella* spp. arteritis	*S*. Dublin
[6]	Aorto-femoral graft	None identified	Phage type 4
[7]	Blalock–Taussig shunt	None identified	*S*. Typhimurium
[9]	Aorto-femoral graft	None identified	*S*. Typhimurium
[2]	Aorto-bifemoral graft	*Salmonella* spp. isolated from urine, presumed to be due to haematogenous spread	*S*. Typhimurium

This case has clear implications for the management of chronic PVGI. Firstly, it is vital to collect high-quality surgical specimens for microbiological analysis in all patients who present with PVGI. It is not possible to devise an empirical regime that would cover all the more unusual pathogens. Since June 2015, prospective surveillance at the Freeman Hospital has revealed that *Staphylococcus aureus*, *S. epidermidis* and *Escherichia coli* are the most common causes of PVGI. However, we have also isolated various organisms that would be difficult to account for with an empirical regimen, including coagulase-negative staphylococci, *Propionibacterium acnes*, *Pseudomonas aeruginosa*, *Corynebacterium striatum* and *Enterococci*.

Secondly, culture of high-quality surgical specimens can inform the choice of antibiotic prophylaxis. *Salmonella* Typhimurium is intrinsically resistant to several commonly used antimicrobials.

Finally, it may be prudent to enquire about a history of acute gastroenteritis when assessing patients presenting with chronic PVGI, and review previous microbiology results. In this case, no faecal specimens were sent for culture between the episode of gastroenteritis, in 2014, and removal of the infected axilla-bifemoral bypass graft, in 2016. Although ‘clearance’ stool cultures are not recommended, this case demonstrates that chronic carriage may present a risk to individuals where haematogenous spread can lead to severe infections, such as PVGI. *Salmonella* Typhimurium was not isolated on culture of a faecal specimen collected post-operatively.

## Learning Points

The underlying anatomy of any prosthetic vascular grafts should be considered in patients presenting with unusual cutaneous abscesses.

A CT scan should be performed before attempting to lance an abscess that might involve an infected prosthetic vascular graft.

It is vital to collect high-quality surgical specimens for microbiological analysis in all patients who present with PVGI.

The choice of empirical antibiotic therapy and surgical prophylaxis should take into account the results of local, prospective surveillance of PVGIs.

Although rare, it is advisable to consider the risk of deep-seated *Salmonella* PVGI infection in any patient who presents with a history of *Salmonella* gastroenteritis, even if this occurred several years ago.
